# Anästhesie bei organtransplantierten Patient:innen

**DOI:** 10.1007/s00101-023-01332-x

**Published:** 2023-10-24

**Authors:** Anna Fiala, Robert Breitkopf, Barbara Sinner, Simon Mathis, Judith Martini

**Affiliations:** Universitätsklinik für Anästhesie und Intensivmedizin, Medizinische Universität Innsbruck, Anichstraße 35, 6020 Innsbruck, Österreich

**Keywords:** Perioperatives Management, Immunsuppression, Monitoring, Prophylaxe, Infektion, Abstoßung, Perioperative management, Immunosuppression, Monitoring, Prophylaxis, Infection, Rejection

## Abstract

Organtransplantierte Patient:innen, die sich einem operativen Eingriff unterziehen müssen, der nicht im Zusammenhang mit ihrer Transplantation steht, können die betreuenden Anästhesist:innen vor große Herausforderungen stellen. Einerseits gilt es, die Transplantatfunktion in der perioperativen Phase sorgfältig hinsichtlich des Auftretens einer etwaigen Abstoßungsreaktion zu überwachen. Andererseits müssen die laufende Immunsuppression ggf. bezüglich des Wirkstoffes und/oder des Applikationsweges den perioperativen Erfordernissen angepasst sowie das sich aus ihr ergebende erhöhte Infektionsrisiko und mögliche Nebenwirkungen (wie z. B. Myelosuppression, Nephrotoxizität, Beeinträchtigung der Wundheilung usw.) in das perioperative Behandlungskonzept integriert werden. Des Weiteren sind möglicherweise persistierende Komorbiditäten der Grunderkrankung sowie physiologische Spezifika infolge der Organtransplantation zu beachten. Hierbei kann auf die Expertise das jeweiligen Transplantationszentrums zurückgegriffen werden.

## Abstract (en)

Organ transplant patients who must undergo nontransplant surgical interventions can be challenging for the anesthesiologists in charge. On the one hand, it is important to carefully monitor the graft function in the perioperative period with respect to the occurrence of a possible rejection reaction. On the other hand, the ongoing immunosuppression may have to be adapted to the perioperative requirements in terms of the active substance and the route of administration, the resulting increased risk of infection and possible side effects (e.g., myelosuppression, nephrotoxicity and impairment of wound healing) must be included in the perioperative treatment concept. Furthermore, possible persistent comorbidities of the underlying disease and physiological peculiarities as a result of the organ transplantation must be taken into account. Support can be obtained from the expertise of the respective transplantation center.

## Einleitung

Im Jahr 2021 wurden im deutschsprachigen Raum mehr als 4700 Organe transplantiert [37]. Eine Organtransplantation verbessert nicht nur das Überleben, sondern auch die Lebensqualität deutlich. Der folgende Artikel bietet einen Überblick über die relevanten Aspekte der perioperativen Betreuung von organtransplantierten Patient:innen. Es werden das allgemeine sowie das (organ‑)spezifische anästhesiologische Management und pathophysiologische Besonderheiten nach den jeweiligen Organtransplantationen erläutert sowie der Umgang mit Immunsuppressiva erklärt.

## Allgemeines anästhesiologisches Management

Bei guter Organfunktion können alle gängigen Allgemein- und Regionalanästhesieverfahren sowie alle Anästhetika verwendet werden, wobei mögliche Wechselwirkungen mit der laufenden Immunsuppression sowie deren mögliche Nebenwirkungen im Hinblick auf Nephro- und Neurotoxizität, Myelosuppression oder Wundheilung zu beachten sind [45]. Vor einem elektiven Eingriff müssen ein Infekt oder eine Abstoßung ausgeschlossen werden [66]. Aufgrund der Immunsuppression besteht ein erhöhtes Risiko für nosokomiale oder opportunistische Infektionen mit z. T. atypischen Krankheitserregern bzw. atypischen klinischen Manifestationen, welche ihrerseits deren Diagnostik erschweren. Große Aufmerksamkeit gilt daher strikter Hygiene – insbesondere bei invasiven Maßnahmen. Zum typischen perioperativen Erregerspektrum kommen seltene opportunistische Erreger bzw. die Reaktivierung latent vorbestehender Infektionen hinzu. Außerdem besteht bei transplantierten Patient:innen häufiger eine antibiotische Vortherapie, und die Patient:innen sind häufiger mit multiresistenten Erregern kolonisiert. Durch die Immunsuppression verlaufen diese Infektionen oft atypisch, und die Diagnostik und Therapie sind erschwert [88].

Das intra- und postoperative Management orientiert sich an der Pathophysiologie der Grunderkrankung und deren Remission nach Transplantation. In Analogie zum Vorgehen bei nichttransplantierten Patient:innen sollten Begleiterkrankungen in das Ausmaß der präoperativen diagnostischen Maßnahmen, des perioperativen Monitorings und der postoperativen Betreuung einfließen.

Die präoperative Diagnostik bei elektiven Eingriffen richtet sich auch bei transplantierten Patient:innen nach den allgemeinen Qualitätsleitlinien, kategorisiert nach Schwergrad des operativen Eingriffs und Co-Morbidität der Patient:innen.

Neben Anamnese und klinischer Untersuchung richtet sich demnach die Notwendigkeit einer individualisierten Risikoevaluierung nach der kardiopulmonalen Leistungsfähigkeit. Eine vorausgegangene Organtransplantation rechtfertigt die Durchführung einer erweiterten Laborchemie, welche neben einer Blutbilduntersuchung auch die Bestimmungen von Blutzucker, Serumelektrolyten und Serumkreatinin beinhalten sollte. Des Weiteren sind transplantierte Patient:innen hinsichtlich ihrer jeweiligen Organfunktion und insbesondere dem etwaigen Auftreten einer Abstoßungsreaktion zu evaluieren.

Abstoßungsreaktionen können auch noch Jahre nach der Transplantation auftreten und gehen zumeist mit unspezifischen Krankheitssymptomen wie Unwohlsein, Kopfschmerz, Schwindel oder Fieber sowie Druckschmerz und Schwellung im Bereich des Transplantates einher.

Je nach transplantiertem Organ ist das Auftreten spezifischer Symptome zu beachten: Rückgang der Urinausscheidung, Zunahme des Körpergewichtes, Hypertonie, Proteinurie/Hämaturie (Niere); Ikterus, Aszites, Enzephalopathie und Koagulopathie (Leber), Beinödeme, Dyspnoe, Zyanose, Nykturie, Hypotonie und Rhythmusstörungen (Herz); Hyperglykämie, Steatorrhö, Diarrhö, Maldigestion und Meteorismus (Pankreas) sowie Dyspnoe (Lunge).

Neben einer sofortigen Spiegelkontrolle der laufenden Immunsuppression sind dann eine organspezifische Laborchemie (Tab. 1), eine Bildgebung und ggf. funktionelle Diagnostik sowie ein entsprechendes Hygienescreening unter Berücksichtigung typischer opportunistischer Krankheitserreger (z. B. Zytomegalievirus [CMV], Epstein-Barr-Virus [EBV], Polyomaviren, *Pneumocystis jirovecii* etc.) erforderlich. Im Falle eines Abstoßungsverdachtes wird eine unmittelbare Kontaktaufnahme mit dem jeweiligen Transplantationszentrum empfohlen; elektive Operationen sollten verschoben werden.

**Table 1:** 

Allgemein	Anamnese, klinische Untersuchung Individualisierte Risikoevaluierung gem. kardiopulmonaler Leistungsfähigkeit Blutbild, Gerinnung, Blutzucker, Elektrolyte, Serumkreatinin
Niere	Kreatinin, Harnstatus Sonographie Flüssigkeitsbilanz
Leber	Bilirubin, Transaminasen, Cholestaseparameter, Gerinnung, Albumin, Ammoniak Sonographie, ggf. CT
Herz	Herzenzyme, NT-pro-BNP EKG, Echokardiographie
Pankreas	HbA_1c_, C‑Peptid, Pankreasenzyme, Stuhlelastase Sonographie, ggf. CT Blutzuckertagesprofil, Insulinbedarf
Lunge	Blutgasanalyse Röntgen, Spirometrie, Sonographie, ggf. CT

Falls perioperativ Blutprodukte verabreicht werden müssen, sollen aufgrund des Risikos von Zytomegalievirus(CMV)-Infektionen bei CMV-negativen Patient:innen ausschließlich leukozytenarme (depletierte) Produkte verwendet werden [33]. Erythrozytenkonzentrate müssen nicht bestrahlt werden [42].

## Anästhesie nach Herztransplantation

### Anatomie und Pathophysiologie

Nach einer Herztransplantation (HTX) besteht der Zustand einer autonomen Denervierung, da die gesamte parasympathische und intrinsische postganglionäre sympathische Innervation zum Myokard unterbrochen ist [33]. Physiologischerweise innervieren parasympathische Fasern (via Acetylcholin) v. a. die Vorhöfe sowie das Reizleitungssystem und bewirken negativ-chronotrope, dromotrope und inotrope Effekte. Sympathische Fasern hingegen sind über das gesamte Herz verteilt und schütten an den postganglionären Synapsen v. a. Noradrenalin und etwas weniger Adrenalin aus [39].

Das transplantierte Herz besitzt zwar seinen eigenen Sinusknoten, dieser bekommt jedoch keine afferenten Impulse mehr, und somit funktionieren beispielsweise der Barorezeptorreflex oder der Karotissinusreflex nicht mehr; auch kommt es nach einem Valsalva-Manöver zu keiner Herzfrequenzänderung, und auch die reflektorische Herzfrequenzsteigerung auf Hypotonie bzw. Hypovolämie ist deutlich verlangsamt [20, 33, 61]. Eine vagale Reflexantwort auf die Laryngoskopie fehlt ebenso wie eine tachykarde Reflexantwort auf eine zu oberflächliche Anästhesie. Fehlende efferente Fasern zum Rückenmark scheinen auch die Ursache dafür zu sein, dass bei kardialen Ischämien keine Angina-pectoris-typischen Schmerzen auftreten [4, 36]. Der Großteil aller HTX-Patient:innen (40–70 %) zeigen zwischen 6 Monaten und 3 Jahren nach der Transplantation eine teilweise autonome Reinnervierung [4, 10], das Ausmaß ist jedoch individuell sehr variabel und nicht vorhersagbar [14, 18].

Der Frank-Starling-Mechanismus bleibt hingegen auch nach einer HTX intakt, da er von der sympathischen oder parasympathischen Innervation unabhängig ist [84]. Hierbei erhöht sich das Herzzeitvolumen durch eine Erhöhung des Schlagvolumens, was die enorme Wichtigkeit eines ausgeglichenen Volumenstatus unterstreicht, um hämodynamische Stabilität zu garantieren [60].

Die Ruheherzfrequenz des transplantierten Herzens liegt bei 90–100 Schlägen/min, da aufgrund der Denervierung der physiologische vagale Ruhetonus fehlt, es aber weiterhin auf zirkulierende oder exogen zugeführte Katecholamine sensibel ist. Deshalb kann es – v. a. während des ersten Jahres nach HTX – zu Herzrhythmusstörungen kommen, die mit einer erhöhten Sensitivität auf Katecholamine zusammenhängen [84].

Die chirurgische Technik hat auch einen Einfluss auf postoperative Rhythmusstörungen. Bei der (häufigeren) bikavalen Technik wird der rechte Spendervorhof mit der V. cava superior und der V. cava inferior des Empfängers anastomosiert, und nur ein kleiner Anteil des linken Vorhofs des Empfängers mit den Mündungen der Pulmonalvenen verbleibt in situ und wird mit dem linken Vorhof des Spenders anastomosiert [3, 61]. Im Vergleich zur (älteren) biatrialen Technik, bei der die Empfängervorhöfe mit den Spendervorhöfen anastomosiert werden, kommt es bei der bikavalen Technik im postoperativen Verlauf zu weniger permanenten Schrittmacherimplantationen [57, 70].

Erstgradige AV-Blockierungen kommen nach einer HTX häufig vor; ebenso entwickeln bis zu 30 % der Patient:innen einen Rechtsschenkelblock. Eine permanente Schrittmacherimplantation benötigen 14 % aller herztransplantierten Patient:innen [71]. Wenn Anteile des Empfängersinusknotens noch in situ verbleiben (v. a. bei der biatrialen Technik), kann das EKG von HTX-Patient:innen 2 P‑Wellen aufweisen. Die elektrische Aktivität vom Empfängersinusknoten wird jedoch (aufgrund der Vorhofnaht) nicht an das nachgeordnete Reizleitungssystem weitergeleitet. Durch die asynchrone Kontraktion der beiden Vorhofanteile kommt es zu einer Reduktion des Schlagvolumens [3].

Im Normalfall zeigt das Spenderherz eine erhaltene systolische Linksventrikelfunktion, sofern es zu keiner akuten Abstoßungsreaktion kommt. Die diastolische Funktion hingegen kann anfänglich deutlich kompromittiert sein, was sich in erhöhten Füllungsdrücken manifestiert [60, 75]. Die dadurch eingeschränkte Ventrikelfüllung macht eine genau dosierte Volumensubstitution – unter Vermeidung einer Hypervolämie – besonders wichtig.

Die „Allograft-Vaskulopathie“ ist eine beschleunigte Vasosklerose der Koronargefäße, die 5 Jahre nach der Transplantation fast 30 % aller Patient:innen betrifft und wesentlich zur Morbidität und Mortalität beiträgt [33, 45, 54].

#### Pharmakologie

Aufgrund der autonomen Denervierung sind *indirekt* wirksame Substanzen, die ihren Effekt über das autonome Nervensystem ausüben, am transplantierten Patienten nicht wirksam. *Direkt *wirkende Substanzen hingegen, die über intrinsische α‑ oder β‑Rezeptoren wirken, sind effektiv. Daher führen die Parasympatholytika Atropin oder Glycopyrrolat nach einer HTX zu keiner Steigerung der Herzfrequenz. Von den gängigen Vasopressoren ist Ephedrin als direkt *und* indirekt wirkendes Sympathikomimetikum weiterhin wirksam. Prinzipiell empfiehlt es sich, nur *direkt* wirksame Substanzen zu verwenden, z. B. Noradrenalin, Epinephrin, Neosynephrin, Dopamin, Dobutamin oder Isoproterenol. Umgekehrt bewirken β‑Blocker auch am transplantierten Herzen eine Senkung der Herzfrequenz.

Im Speziellen erwähnt werden müssen Substanzen, die zur Reversierung der neuromuskulären Blockade verwendet werden. Obwohl Neostigmin eine indirekt wirkende Substanz ist und somit am denervierten transplantierten Herzen unwirksam sein sollte, wurden schwere Bradykardien und sogar Asystolien nach Verabreichung von Neostigmin/Glycopyrrolat beschrieben, v. a. wenn die Transplantation bereits > 6 Monate zurücklag [9, 11, 74]. Hierbei scheinen die parasympathische Reinnervierung sowie direkte vagotone Effekte von Neostigmin an den postganglionären muskarinergen Rezeptoren eine Rolle zu spielen [5, 6, 72]. Allerdings lag bei allen beschriebenen Fällen von Bradykardien oder Asystolien eine kardiale Pathologie zugrunde, die eine derartige Reaktion begünstigen könnte (KHK, Abstoßungsreaktion, Sinusknotendysfunktion) [9, 11, 12, 74]. Eine jüngere retrospektive Untersuchung zeigte keine negativen Auswirkungen von Neostigmin/Glycopyrrolat bei HTX-Patient:innen [8]. Manche Autoren empfehlen Sugammadex zur Reversierung einer neuromuskulären Blockade [87]. Doch auch unter Sugammadex wurden Bradykardien und sogar Asystolien beschrieben [69, 73, 96]. Zusammenfassend können zur Reversierung sowohl Neostigmin/Glycopyrrolat als auch Sugammadex verwendet werden; die Verabreichung sollte jedoch mit großer Vorsicht und unter Bereitschaft eines direkten Sympathikomimetikums (z. B. Isoproterenol) erfolgen.

#### Spezifische anästhesiologische Überlegungen nach HTX

Die V. jugularis interna (rechts) ist die bevorzugte Stelle für regelmäßig erforderliche Biopsien und sollte, wenn möglich, geschont werden. Bei zu erwartendem größeren Blutverlust sollte großzügig ein erweitertes hämodynamisches Monitoring (z. B. mittels Pulmonalarterienkatheter (PAK) oder PiCCO®) eingesetzt werden, das auch während der postoperativen Phase genutzt werden kann, um eine hämodynamische Instabilität zu erfassen. Hierbei richtet sich die Indikation für ein erweitertes Monitoring nach den aktuellen ESC Guidelines für nichtherzchirurgische Eingriffe [30]. Für die intraoperative Evaluierung des Volumenstatus oder der Kontraktilität (v. a. auch der Rechtsventrikelfunktion) eignet sich die transösophageale Echokardiographie (TEE), die grundsätzlich großzügig eingesetzt werden sollte, wenn es zu intraoperativer hämodynamischer Instabilität kommt [41]. Die Indikation für Klebe-Defibrillationselektroden richtet sich nach der Anamnese bzw. sollte erfolgen, wenn ein implantierbarer Kardioverter-Defibrillator (ICD)-Schrittmacher intraoperativ deaktiviert wird.

Besondere Aufmerksamkeit muss auf ein ausgeglichenes Volumenmanagement und auf die Aufrechterhaltung einer adäquaten Herzfrequenz gelegt werden, um das Herzzeitvolumen im Normbereich zu halten. Ebenso sollten Medikamente zur Blutdrucksteigerung, Herzfrequenzsteigerung und evtl. zur Unterstützung der Inotropie vorgehalten werden.

Bei Patient:innen mit ICD-Schrittmachern soll je nach Hausstandard (intraoperative Magnetauflage/Umprogrammieren und postoperatives Rückprogrammieren) verfahren werden.

## Anästhesie nach Lungentransplantation

Die Transplantation der Lunge (LuTX) ist heute eine Standardbehandlung für zahlreiche Lungenerkrankungen im Endstadium wie z. B. chronisch obstruktive Lungenerkrankungen (COPD), Lungenfibrose, zystische Fibrose und pulmonale Hypertension.

### Anatomie/Operationstechnik

Die klassische „En-bloc-Technik“ mit einer einzigen Trachealanastomose wird nicht mehr durchgeführt [31]. Heute werden sowohl Einzel- (SLuTX) als auch Doppellungentransplantationen (DLuTX) sequenziell mit separaten bronchialen, pulmonalarteriellen und pulmonalvenösen Anastomosen durchgeführt [13].

### Atemphysiologie/Beatmung

Durch die LuTX kommt es zur Unterbrechung der pulmonalen Innervation, des Lymphabflusses und der bronchialen Durchblutung. Der geschädigte Lymphabfluss prädisponiert (insbesondere in der Frühphase) für ein Lungenödem [49, 66, 78]. Bei Patient:innen, die noch mit der alten „En-bloc-Technik“ operiert wurden, können – im Gegensatz zur LuTX mit erhaltener Empfänger-Carina – Sensibilität und Hustenstoß distal der Trachealanastomose komplett erloschen sein [49, 66].

Lungentransplantierte Menschen haben eine eingeschränkte mukoziliäre Clearance sowie eine höhere Inzidenz von Motilitätsstörungen des Ösophagus sowie gastroösophagealer Refluxkrankheit [33, 45]. Sie sind somit prädispositioniert für (stille) Aspirationen, Sekretretentionen („mucus plugging“) und Infektionen [42, 66].

Besonders SLuTX-Empfänger:innen können herausfordernd zu beatmen sein: Liegt bei der patienteneigenen Lunge ein Emphysem vor, so besteht bei Beatmung die Gefahr einer dynamische Hyperinflation mit dem Risiko für einen Spannungspneumothorax oder auch für einen Mediastinalshift mit Kompression der transplantierten Lunge [22, 33, 42, 78]. Eine Restriktion der nativen Lunge kann im Gegensatz dazu erhöhte Beatmungsdrücke erforderlich machen, die bei der transplantierten Lunge zu Volu- und Barotraumen führen können [42, 45]. Zur Optimierung der Beatmungssituation kann in diesen Fällen ein Intensivbeatmungsgerät oder eine seitengetrennte Ventilation erwogen werden [33, 78]. Unterschiedliche Compliances zwischen den beiden Lungenflügeln nach SLuTX können zu biphasischem endtidalen Kohlenstoffdioxid (CO_2_) führen [31].

Werden Patient:innen nach SLuTX einem Eingriff mit Einlungenventilation unterzogen, so ist das Risiko einer Hypoxämie besonders hoch, wenn die transplantierte Lunge, welche 60–80 % der Ventilation und Perfusion erhält, nicht ventiliert wird [33, 45]. In diesem Fall kann eine zusätzliche Jet-Ventilation der zu operierenden transplantierten Lunge versucht werden.

Patient:innen nach DLuTX sind hingegen meist unkomplizierter zu beatmen. Wie bei nichttransplantierten Patient:innen wird eine lungenprotektive Beatmung empfohlen [22, 33, 78].

Eine Larynxmaske ist in Abhängigkeit vom Aspirationsrisiko für kurze, elektive Eingriffe möglich [22]. Um gut endotracheal absaugen oder bronchoskopieren zu können, sollte der größte Tubus, der atraumatisch platziert werden kann, verwendet werden [42]. In-vitro-Daten zeigen, dass bei schlechter Compliance während der Bronchoskopie auch durch große endotracheale Tuben (ETT) (Gr. 8,0) hohe Plateaudrücke und ein Auto-Peep generiert werden. Bei normaler Compliance wird dagegen auch bei einem ETT der Gr. 6,0 kein Spitzendruck > 30 cm H_2_0 beobachtet, sofern ein kleines Bronchoskop verwendet und ein verlängertes Inspirium gewährleistet wird [46]. Für eine seitengetrennte Beatmung können ein Doppellumentubus oder ein Bronchusblocker verwendet werden [33]. Falls ein Doppellumentubus über die bronchiale Anastomose oder eine endotracheale Intubation über die Carina-Anastomose (bei nach „En-bloc-Technik“ operierten Patient:innen) erforderlich ist, empfiehlt sich ein bronchoskopisches Einführen, um Verletzungen der Anastomosen zu vermeiden [33, 42].

### Begleiterkrankungen

Die 5‑Jahres-Morbidität zeigt eine hohe Prävalenz von arterieller Hypertension (> 80 %), Hyperlipidämie (> 50 %), Niereninsuffizienz (37 %), Diabetes mellitus (35 %) und Bronchiolitis-obliterans-Syndrom (BOS, 33,7 %) sowie Osteoporose und Malignomen [31, 78].

Früh spielen Infektionen und Abstoßung eine Rolle, das Langzeit-Outcome wird durch „community-acquired infectious diseases“, Neoplasmen, lymphoproliferative Erkrankungen und das BOS bestimmt [22]. BOS ist eine Form der chronischen Abstoßung – histologisch zeigen sich eine chronische Entzündung, Fibrose und Obliteration der kleinen Atemwege [33, 42, 45]. Klinische Zeichen sind trockener Husten, Dyspnoe und Abgeschlagenheit. Differenzialdiagnosen sind Infektionen bzw. Pneumonien [22, 49, 66]. Begleitende Bronchiektasien prädisponieren zu Infektionen mit gramnegativen Erregern (*Pseudomonas*) oder Aspergillen [22].

Präoperativ soll eine ausführliche Anamnese erfolgen – insbesondere bezüglich Dyspnoe, Husten, Sputum, Belastbarkeit, Sauerstoffbedarf sowie rezidivierenden Infekten/Abstoßungsepisoden [33, 45]. Nach einer DLuTX zeigt sich eine nahezu vollständige Normalisierung der Lungenfunktionstests [45]. Bei einer Verschlechterung sollen Abstoßung, BOS oder Infektion präoperativ ausgeschlossen werden [33]. Eine persistierende Hyperkapnie kann durch eine Graft-Dysfunktion oder eine Phrenikusparese verursacht sein, wogegen eine akuter (Wieder‑)Anstieg des arteriellen Kohlenstoffdioxid-Partialdrucks (p_a_CO_2_) Hinweis auf eine akute Abstoßung oder Infektion sein kann [31, 45]. Jede Verschlechterung sollte zu einer Kontaktaufnahme mit dem zuständigen Transplantationszentrum veranlassen [42].

### Spezifische anästhesiologische Überlegungen nach LuTX

Die Indikation zu einer invasiven arteriellen Blutdruckmessung (inklusive BGA) wird meist großzügig gestellt [66]. Aufgrund der erhöhten Empfindlichkeit gegenüber Volumenüberladung mit der Gefahr eines Lungenödems kann ein erweitertes Monitoring zur Herzzeitvolumenmessung bzw. Abschätzung des Volumenstatus indiziert sein. Dabei sollte – wie bei jedem/r Patient:in – eine möglichst wenig invasive Methode wie die TEE oder ein noninvasives hämodynamisches Monitoring mit Pulskonturanalyse (z. B. PiCCO® zur Abschätzung des extravaskulären Lungenwassers) gewählt werden [22, 33, 42]. Ein Pulmonalarterienkatheter (PAK) sollte aufgrund der Invasivität nur bei spezifischen Fragenstellungen (pulmonale Hypertonie) zum Einsatz kommen.

Regionalanästhesieverfahren können Vorteile bieten, da keine Atemwegsmanipulation und keine Beatmung nötig sind [22, 33, 49, 66]. Eine effiziente postoperative Schmerztherapie ist essenziell, um effektives Abhusten, suffiziente Sekret-Clearance und frühe Mobilisation zu ermöglichen [22]. Trotz einer durch die Immunsuppression erhöhten Infektanfälligkeit ist keine erhöhte Rate an infektiösen Komplikationen nach Regionalanästhesie beschrieben [33]. Jedoch kann eine iatrogene Zwerchfellparese oder Schwächung der Atemhilfsmuskulatur durch neuroaxiale Verfahren Lungentransplantierte respiratorisch deutlicher beeinträchtigen als lungengesunde Patient:innen [22, 33].

## Anästhesie nach Lebertransplantation

### Anatomie/Pathophysiologie

Das Transplantat wird bei der orthotopen Lebertransplantation (LTX) nach dem Entfernen des erkrankten Organs an dieselbe Stelle im rechten Oberbauch anastomosiert. Für nachfolgende Oberbaucheingriffe erhöhen evtl. vorhandene extraanatomische Gefäßrekonstruktionen oder biliodigestive Anastomosen die Gefahr von Blutungen und Organverletzungen. Unmittelbar nach der LTX sinkt der portalvenöse Widerstand deutlich und nimmt in den ersten Jahren weiter ab [32]. Da die portale Hypertension die Ursache für viele anästhesierelevante Komorbiditäten (z. B. Ösophagusvarizen, Aszites) ist, bilden sich diese Erkrankungen normalerweise mit dem Rückgang der portalen Hypertension ebenfalls zurück [2, 38]. Auch die hyperdynamische Kreislauflage normalisiert sich nach der Transplantation.

Neben klinischen Zeichen der Transplantatinsuffizienz (Ikterus, Knöchelödeme, Aszites, Juckreiz) sollten vor jedem chirurgischen Eingriff Laborparameter, die auf Leberpathologien hinweisen, ermittelt werden (Tab. 1). Obwohl ein Großteil der Transplantate später histologische Auffälligkeiten aufweist, die in diesen Laborwerten nicht abgebildet werden, kann angenommen werden, dass unauffällige Leberfunktionsparameter für eine ausreichende Leistung des Transplantats sprechen [76, 77]. Finden sich Anzeichen für eine Abstoßungsreaktion, Transplantatinsuffizienz oder eine Abflussstörung der Gallenwege, sollten nach Möglichkeit im Vorfeld der Operation eine Abklärung und Therapie im Zentrum veranlasst werden. Bei eingeschränkter Syntheseleistung des Organs zeigt sich eine (zumindest laborchemisch) eingeschränkte Gerinnung. Dadurch kann eine Kontraindikation für Regionalanästhesien bestehen.

### Spezifische anästhesiologische Überlegungen nach LTX

Intraoperativ können Residuen der portalen Hypertension herausfordernd sein: Normalerweise beginnen Ösophagusvarizen bereits unmittelbar nach der Transplantation durch den Abfall des portalvenösen Widerstandes deutlich zu schrumpfen und bleiben im Weiteren nur bei wenigen Patienten nachweisbar [47]. Trotzdem sollte, besonders in den ersten Monaten nach LTX sowie nach der Transplantation von Split-Organen, mit nach wie vor vorhandenen Ösophagusvarizen gerechnet werden und die Indikation für Magensonden und die TEE entsprechend streng gestellt werden. In seltenen Fällen, die jedenfalls weiterer Abklärung bedürfen, leiden Patient:innen nach einer LTX weiterhin unter Aszites [27]. Ursachen dafür können beispielsweise abdominelle Infektionen oder eine Obstruktion der V. portae sein.

Große Aszitesmengen gehen mit einer Reduktion der funktionellen Residualkapazität und dadurch mit einer schnellen Sauerstoffdesaturation bei der Narkoseeinleitung einher. Außerdem ergeben sich durch die Hypoalbuminämie, die Zunahme des extrazellulären Raumes und das dadurch erhöhte Verteilungsvolumen für Medikamente Konsequenzen für die Narkoseeinleitung.

Ein Großteil der Patient:innen mit hepatopulmonalem Syndrom (HPS), dessen Grund v. a. intrapulmonale Shunts sind, erfährt während des ersten Jahres nach der LTX eine vollkommene Remission [62, 85]. Dabei kann in seltenen Fällen eine pulmonalarterielle Hypertonie, die vor der Transplantation durch das klinisch im Vordergrund stehende HPS nicht relevant war, demaskiert werden [68]. Hierbei sollte ein entsprechendes Monitoring (Pulmonaliskatheter) in Betracht gezogen werden. Bei Symptomen, die auf eine Persistenz des HPS hinweisen, ist die Indikation zur Durchführung einer präoperativen Blutgasanalyse großzügig zu stellen und evtl. die postoperative Betreuung auf einer Intensivstation sicherzustellen [62].

Ein hepatorenales Syndrom bleibt bei etwa 10 % der Patienten auch nach der Lebertransplantation bestehen [15, 81]. Außerdem besteht oft eine Einschränkung der Nierenfunktion anderer Genese. Dies sollte durch die Bestimmung der Retentionsparameter erkannt und das anästhesiologische Management sowie die verwendeten Medikamente dementsprechend angepasst werden. Intraoperativ ist v. a. auf einen ausreichenden Perfusionsdruck zu achten, der sich an den physiologischen Werten orientieren soll. Bei normaler Leberfunktion können alle zur Verfügung stehenden Medikamente verabreicht werden. So kann beispielsweise auch Paracetamol zur postoperativen Analgesie verwendet werden [21]. Sollte die Leberfunktion eingeschränkt sein, muss von einer herabgesetzten Clearance mit verlängerter Wirkdauer hepatisch eliminierter Medikamente gerechnet werden. Besonders davon betroffen sind Muskelrelaxanzien (mit Ausnahme von Atracurium und Cisatracurium), deren Wirkung mithilfe der Relaxometrie überwacht werden sollte. Bei der Verwendung von inhalativen Narkotika wird v. a. Sevofluran empfohlen [83]. Um die Rheologie im transplantierten Organ aufrechtzuerhalten und Thrombosen in der A. hepatica entgegenzuwirken, ist eine Dehydration zu vermeiden.

## Anästhesie nach Nierentransplantation und Nieren-Pankreas-Transplantation

### Nierentransplantation

#### Anatomie und Pathophysiologie

Die Niere wird heterotop im Bereich der linken oder rechten Fossa iliaca transplantiert. Der arterielle Zu- und Abfluss erfolgen über die A. bzw. V. iliaca. Im Laufe der Zeit und in Abhängigkeit von Abstoßungsreaktionen lässt sich eine zunehmende Fibrosierung beobachten. Für die transplantierte Niere bedeutet dies nicht nur, dass die Funktion nachlässt, sondern auch, dass die Arterie zunehmend steif wird und möglicherweise stenosiert, was mit einem erhöhten Gefäßwiderstand verbunden ist [1, 65].

### Pankreastransplantation

#### Anatomie und Pathophysiologie

Eine Pankreastransplantation (PTX) wird selten allein, sondern meistens in Kombination mit einer Nierentransplantation (NTX) als NPTX durchgeführt. Die häufigste Indikation stellt der Diabetes mellitus (DM), gefolgt von der zystischen Fibrose, dar. Ein DM geht im Laufe der Zeit häufig mit einer Niereninsuffizienz einher, weshalb bei einer Indikation zur PTX in der Regel auch die Indikation zur NTX besteht. Das Pankreas wird meist kontralateral zur Niere transplantiert und ebenfalls an der A. iliaca angeschlossen. Der venöse Abstrom erfolgt entweder über die V. iliaca oder die V. portae. Der Pankreasgang wird – meist über eine Y‑Roux-Schlinge – an das Ileum angeschlossen.

#### Begleiterkrankungen

Aufgrund der Grunderkrankung und ggf. langer Dialysezeit leiden Patienten nach (P)NTX häufig an kardiovaskulären Erkrankungen, und diese stellen auch die häufigste Todesursache (50–60 %) dar [44, 64]. Die Begleiterkrankungen bei PTX sind in der Regel die typischen Begleiterkrankungen des DM.

Bei den meisten (50–80 %) aller (P)NTX Patient:innen liegt eine arterielle Hypertension vor. Neben der Grunderkrankung können hierfür Kalzineurin-Inhibitoren (CNI), Glukokortikoide, eine Dyslipidämie, Adipositas und eine durch Salzretention und Vasokonstriktion bedingte Gewichtszunahme verantwortlich gemacht werden [17, 26, 43]. Für die Entwicklung eines DM nach NTX sind der schnelle Metabolismus und die Ausscheidung des Insulins durch die transplantierte Niere, die Gluconeogenese der transplantierten Niere und die Immunsuppressiva verantwortlich [50, 97].

Anämie, v. a. im ersten Jahr nach einer Transplantation (TX), ist mit verminderter Transplantatfunktion und erhöhter Mortalität verbunden [23]. Sie kann als Folge der Niereninsuffizienz, Einnahme von Immunsuppressiva, Virostatika, Infektionen, Einnahme von Agiotensin-Converting-Enzyme(ACE)-Rezeptor-Antagonisten bzw. Angiotensin-II(AT2)-Rezeptor-Blockern auftreten [16, 56, 79].

Bei einer Posttransplantationserythrozytose handelt es sich um eine dauerhafte Erhöhung des Hämatokrits über 51 %. Sie tritt in 10–15 % der Fälle auf und entwickelt sich üblicherweise nach 8 bis 24 Monaten [90]. Ursächlich wird u. a. eine persistierende Erythropoetinsekretion durch die verbliebene, afunktionelle Niere und fehlende Feedbackregulation diskutiert. Die Therapie besteht aus ACE-Hemmern oder AT2-Rezeptor-Blockern [90].

Als Folge nachlassender Nierenfunktion bzw. der Immunsuppression werden Störungen des Elektrolyt- und Säure-Basen-Haushalts wie Hyperkaliämie und Hyperkalzämie, Hypophosphatämie und Hypomagnesiämie sowie eine metabolische Acidose beobachtet. Der gestörte Knochen- und Kalziumstoffwechsel ist häufig mit Osteoporose, Hyperparathyreoidismus und Vitamin-D-Mangel assoziiert.

Im Rahmen der Anamnese und präoperativen Abklärung (Tab. 1) sollte besonderes Augenmerk auf kardiovaskuläre Begleiterkrankungen und die Folgen des DM gelegt werden. Normale Blutzuckerkonzentrationen zeigen eine gute Organfunktion an. Ein DM kann trotz guter Pankreasfunktion entstehen, weil sich Autoantiköper gegen die neuen Inselzellen bilden können [89]. Die Serum- und Urinamylase stellen gute Parameter für die Einschätzung einer Abstoßungsreaktion dar [67].

#### Spezifische anästhesiologische Überlegungen nach *N*(P)TX

Bei eingeschränkter Nierenfunktion sollte eine Dosisanpassung der renal eliminierten Medikamente erfolgen. Gegebenenfalls kann auf kürzer wirksame Medikamente oder Medikamente, die nicht renal eliminiert werden, zurückgegriffen werden. Cholinesteraseinhibitoren wie Neostigmin und Pyridostigmin werden ausschließlich renal eliminiert. Aus theoretischen Überlegungen kann bei Nierenversagen Sugammadex verwendet werden, wobei die Datenlage hierfür schwach ist [48]. Für die Schmerztherapie sollte auf nichtsteroidale Antirheumatika (NSAR) verzichtet werden, weil diese aufgrund der Vasokonstriktion in der Niere zu einer Verschlechterung der Filtrationsleistung führen.

Da sowohl Niere als auch Pankreas druckpassiv perfundiert werden, empfiehlt es sich, das Blutdruckmanagement am Ausgangsblutdruck und an den Komorbiditäten zu orientieren [51]. Bei Anlage einer invasiven Blutdruckmessung an der oberen Extremität muss beachtet werden, dass die A. radialis evtl. zu einem späteren Zeitpunkt für die Anlage eines Cimino-Shunts benötigt werden könnte. Beim Atemwegsmanagement sollte, je nach Vorerkrankungen wie z. B. DM, mit verzögerter Magenentleerung gerechnet werden. Hier hilft ein Ultraschall des Magens, um die Magenfüllung zu beurteilen und ggf. eine Ileuseinleitung („rapid sequence induction“) durchzuführen [52]. Aufgrund eines „stiff joint“ muss bei DM gehäuft mit einem schwierigeren Atemweg gerechnet werden [92].

## Immunsuppressiva

Die Dauertherapie mit Immunsuppressiva sollte bei Patient:innen nach Organtransplantationen perioperativ grundsätzlich unter regelmäßiger Spiegelkontrolle fortgeführt werden, wobei sich das Kontrollintervall nach der zugrunde liegenden Erkrankung bzw. Art und Schwere des operativen Eingriffs richtet [53, 66, 91]. Davon ausgehend, dass die Dosierung der Immunsuppressiva vom betreuenden Zentrum unter regelmäßiger Spiegelkontrolle für die Erhaltungsphase bei stabiler Transplantatfunktion vorgegeben wurde, sind Spiegelkontrollen v. a. beim klinischen Verdacht einer Abstoßung oder beim Eintreten einer medikamentösen Nebenwirkung sowie spiegelbeeinflussenden Erkrankungen (wie z. B. im Rahmen von Infekten und gastrointestinalen Erkrankungen usw.), bei Veränderungen der Serumproteinkonzentration bzw. dem Einsatz von Medikamenten mit entsprechenden pharmakokinetischen Wechselwirkungen angezeigt.

Neben Präparaten zur Induktions- oder Abstoßungstherapie (z. B. T‑Zell-depletierende poly-/monoklonale Antikörper oder der gegen den Interleukin-2-Rezeptor gerichtete Antikörper Basiliximab), basiert die Erhaltungstherapie im Wesentlichen auf einer variierenden Kombination von 2 bis 3 Präparaten aus den im nächsten Abschnitt beschriebenen Substanzklassen [35, 55]. Mögliche Wechselwirkungen limitieren den Einsatz einiger Medikamentengruppen. Allgemein liegen nur begrenzte wissenschaftliche Daten zu den Auswirkungen einer Narkose auf die Immunsuppression vor.

### Kortikosteroide

Kortikosteroide beeinflussen die Aussendung von Zytokinen und anderen immunologischen Mediatoren. Dadurch verhindern sie, dass sich neue Immunzellen bilden und vermehren. Sie werden vorwiegend direkt postoperativ und zur Therapie akuter Abstoßungsreaktionen eingesetzt und meist im Langzeitverlauf in der Dosis reduziert bzw. komplett ausgeschlichen [55]. Die wesentlichen Nebenwirkungen einer Steroidtherapie sind u. a. Hypertonie, Hyperglykämie, Hypokaliämie, Osteoporose, erhöhtes Thromboserisiko, Myopathie, peptische Ulzera und Psychosen. Bei Langzeiteinnahme muss perioperativ mit dem Auftreten einer Nebennierenrindeninsuffizienz gerechnet werden. Bei einer Dosierung über der Cushing-Schwelle wird eine perioperative Substitution empfohlen. Als Mittel der Wahl gilt Hydrocortison, da dies in höherer Dosierung neben der gluko- auch eine ausreichende mineralokortikoide Wirkung aufweist. Die Dosierung richtet sich nach dem Schweregrad der Operation, zumeist werden am Operationstag 100 mg Hydrocortison i.v. anstatt der oralen Kortikoidmedikation verabreicht, welche postoperativ ehestmöglich wieder fortgesetzt werden sollte. Gegebenenfalls kann es notwendig sein, die i.v.-Substitution für einen bis 2 weitere Tage fortzuführen, hierbei wird die initiale Hydrocortisondosis zumeist um täglich jeweils 25 mg reduziert, bis die Dauermedikation wieder fortgeführt werden kann [24].

### Kalzineurininhibitoren

Zu den Kalzineurininhibitoren (CNI) gehören die Substanzen Ciclosporin (Cyclosporin A, CyA) und Tacrolimus (TAC). Sie verhindern – über einer Hemmung der kalzium- und calmodulinabhängigen Proteinphosphatase Kalzineurin – die Aktivierung von NFAT (Nuclear factor of activated T cells) und über eine Zytokinregulation letztlich die IL-2-abhängige Aktivierung von T‑Zellen [55]. CNI werden nicht miteinander kombiniert, bilden aber die Grundlage der meisten Behandlungsprotokolle mit interindividuell stark variierenden Zielplasmaspiegeln.

Neben den allgemeinen metabolischen Nebenwirkungen (Hypertonie, Hyperlipidämie, Hyperglykämie, Hyperkaliämie, Hypomagnesiämie, Hyperurikämie) stehen perioperativ v. a. das nephro-, hepato-, myelo- und neurotoxische Risikopotenzial im Vordergrund. Selten kann TAC eine hypertrophe Kardiomyopathie auslösen und durch eine Verlängerung der QT-Zeit Torsades de Pointes hervorrufen [34]. Als weitere seltene Nebenwirkungen von CNI sind die Entwicklung eines posterioren reversiblen Enzephalopathiesyndroms oder eines atypischen hämolytisch-urämischen Syndroms bekannt [40, 63].

Sowohl TAC als auch CyA haben ein hohes Wechselwirkungspotenzial, da sie einer intensiven Zytochrom-P450-abhängigen (CYP) Oxidation unterliegen. Wenn starke CYP3A4-Inhibitoren (z. B. Metoclopramid, Verapamil, Diltiazem, Azolantimykotika, Makrolide u. a.) oder CYP3A4-Induktoren (z. B. Barbiturate, diverse Tuberkulostatika, Antikonvulsiva u. a.) kombiniert verabreicht werden, sind engmaschige Kontrollen erforderlich: Erhöhte Spiegel gehen mit einer erhöhten Toxizität (v. a. Nephrotoxizität) bzw. erniedrigte Spiegel mit einem erhöhten Abstoßungsrisiko einher.

CyA ist ein Inhibitor von CYP3A4, des Multidrug-Efflux-Transporters P‑Glykoprotein und der Organo-Anion-Transporter und kann die Plasmaspiegel von Begleitmedikationen erhöhen, die Substrate dieses Enzyms und/oder der Transporter sind (z. B. Statine, Dabigatran). Amiodaron, mit seiner sehr langen Halbwertszeit von ca. 50 Tagen, erhöht bei gleichzeitigem Anstieg des Serumkreatinins die CyA-Plasma-Konzentration noch lange Zeit nach dem Absetzen von Amiodaron. Octreotid vermindert die orale Absorption von CyA. Bei gleichzeitiger Anwendung von Diclofenac und CyA wurde ein signifikanter Anstieg der Bioverfügbarkeit von Diclofenac mit dem Risiko einer Nierenfunktionsbeeinträchtigung beschrieben [58].

Galenisch ist der Wirkstoff CyA als Weichgelatinekapsel, Trinklösung oder Konzentrat zur Infusionsbereitung verfügbar und kann daher perioperativ problemlos 1:1 enteral sondiert bzw. 3:1 parenteral substituiert werden. Die Infusionslösung enthält polyethoxyliertes Rizinusöl, welches bei parenteraler Applikation anaphylaktische Reaktionen auslösen kann. Gemäß Hersteller beträgt die empfohlene parenterale Dosis ca. ein Drittel der oralen Dosierung. Das Konzentrat zur Herstellung einer Infusionslösung ist hierbei 1:20 bis 1:100 mit normaler Kochsalzlösung oder 5 %iger Glucoselösung zu verdünnen und als langsame i.v.-Infusion über 2–6 h zu verabreichen. Vom Wirkstoff TAC sind mittlerweile neben den Immediate-Release-Präparaten (2-mal tägliche Gabe) auch zwei unterschiedliche Retard-Präparate (einmal tägliche Gabe) verfügbar. Hierbei gilt es zu beachten, dass bei einem hiervon (Envarsus®) die orale Bioverfügbarkeit mittels spezieller Herstellung optimiert wurde. Dabei wird der Wirkstoff geschmolzen auf Trägerpartikel aufgesprüht, die anschließend agglomeriert als Granulat verabreicht werden, wodurch die Tagesdosierung gegenüber den anderen oralen Präparaten um bis zu 30 % reduziert werden kann [7]. Auch bei TAC stehen neben Hartkapseln und Retardtabletten ein Granulat zur Herstellung einer 2‑mal täglich zu verabreichenden Suspension bzw. ein Konzentrat zur Herstellung einer Infusionslösung zur Verfügung. Im Falle einer enteralen Applikation muss ein polyvinylchloridfreies Sondenmaterial sichergestellt werden [86]. Weiters gilt zu beachten, dass die TAC-Resorption in hohem Maße von der Form der enteralen Ernährung bzw. gastrointestinalen Funktion abhängt (kontinuierliche vs. Bolusernährung bzw. Diarrhö) [59]. Die bukkale Applikation einer magistral produzierten Suspension bietet sich an, wenn die intestinale Resorption erheblich beeinträchtigt ist [25]. Bei parenteraler Gabe soll ca. ein Fünftel der oralen Tagesdosierung als Infusion in einer 1:10- bis 1:250-Verdünnung in normaler Kochsalzlösung oder 5 %iger Glucoselösung über 24 h verabreicht werden, wobei im Falle der vorherigen Verwendung jenes Retard-Präparates mit optimierter Bioverfügbarkeit eine entsprechende Dosisanpassung zu beachten ist.

Generell gilt für die CNI, dass aufgrund deren langen Halbwertszeit eine Unterbrechung bis zu 24 h bedenkenlos möglich ist. Die Plasmaspiegel von Patient:innen, die diese Medikamente erhalten, müssen während der perioperativen Phase dennoch täglich überwacht werden. Signifikante Reduktionen der CNI-Spiegel können durch Verdünnung mit massiver Flüssigkeitsinfusion perioperativ und durch kardiopulmonalen Bypass verursacht werden [94]. Bei Patient:innen, die ihre orale CyA-Dosis weniger als 4 h vor der Operation erhielten, wurden über subtherapeutische Spiegel berichtet [93]. Daher wird eine Einnahme 4–7 h vor Operationsbeginn empfohlen. CyA scheint die Wirkung von Muskelrelaxanzien zu verlängern, weshalb bei der Verwendung von Muskelrelaxanzien ein neuromuskuläres Monitoring und ggf. eine Dosisreduktion empfohlen werden [19, 28, 29, 80, 95]. Im Zweifel oder bei Änderungen von Wirkstoffen oder Zieltalspiegeln sollte mit dem jeweiligen Transplantationszentrum rückgesprochen werden.

### mTOR-Inhibitoren

Die mTOR-Inhibitoren Sirolimus und Everolimus verhindern eine Vermehrung der T‑Lymphozyten, indem sie deren Zellteilungszyklus unterbrechen [55]. Sie werden zumeist erst zu einem späteren Zeitpunkt in der Erhaltungstherapie zum Einsatz gebracht, um in Kombination mit TAC die CNI-induzierten Nebenwirkungen zu reduzieren oder überhaupt eine CNI-freie Immunsuppression zu ermöglichen.

In ihrem Nebenwirkungsprofil ähneln sie im Hinblick auf Myelosuppression, Leberfunktionsstörung und Stoffwechselstörungen den CNI, jedoch sind auch Fälle von interstitiellen Lungenerkrankungen sowie postoperative Wundheilungsstörungen mit dem gehäuften Auftreten von Faszien- und Anastomosendehiszenzen sowie Narbenhernien bekannt. Daher wird empfohlen, mTOR-Inhibitoren perioperativ ab 2 bis 5 Tage vor elektiven Operationen bis zum Abschluss der Wundheilung (ca. 10 bis 14 Tage postoperativ) zu pausieren bzw. in Rücksprache mit dem Transplantationszentrum überbrückend auf einen CNI zu wechseln.

### Antiproliferativa

Die Wirkstoffe Mycophenolat-Mofetil (MMF) und Azathioprin (AZT) führen über Hemmung der Inosinmonophosphat-Dehydrogenase bzw. als Purinantagonist zur Hemmung der Lymphozytenproliferation [55].

In der Folge kommt es auch zu einer Myelosuppression sowie teratogenen und gastrointestinalen Nebenwirkungen (inkl. Hepatitis und Pankreatitis). Vor allem unter Antiproliferativa kann eine ausgeprägte Thrombozytopenie auftreten, welche das Blutungsrisiko für rückenmarknahe Regionalverfahren erhöht [33].

AZT darf nicht mit Allopurinol kombiniert werden, da die hierdurch vermehrte Bildung von 6‑Thioguanin-Nukleotiden eine schwere Myelosuppression induzieren kann. Ein perioperatives Absetzen von AZT kann bei Patient:innen, die unter oraler Antikoagulation mit Warfarin stehen, Blutungen auslösen [82].

MMF steht galenisch als Filmtablette, Hartkapseln, orale Suspension oder Pulver zur Herstellung eines Infusionslösungskonzentrats zur Verfügung. Die parenterale Applikation kann dosisident zur oralen Dosierung in einer Konzentration von 6 mg/ml in 5 % Glucoselösung über 2 h erfolgen. AZT wird als Filmtablette vertrieben, kann aber mit Wasser als Suspension oder bei magistraler Produktion einer Injektionslösung parenteral verabreicht werden.

## Fazit für die Praxis

- Bei Patient:innen nach einer Organtransplantation soll sich das perioperative Management an der Grunderkrankung, deren Pathophysiologie und den Begleiterkrankungen orientieren.
- Besondere Beachtung gilt dem erhöhten Infektionsrisiko und strikten Hygienemaßnahmen.
- Bei guter Organfunktion können alle gängigen Anästhesieverfahren und Medikamente angewendet werden.
- Die Immunsuppressiva müssen perioperativ weiterverabreicht werden. Gegebenenfalls ist eine Kortisonstressdosis erforderlich (Cushing-Schwelle).
- Perioperative Plasmaspiegelbestimmungen der Immunsuppressiva sollen erfolgen – insbesondere, wenn mit einer Beeinträchtigung der Resorption oder mit größeren Volumenveränderungen gerechnet werden muss. Gegebenenfalls müssen alternative Applikationswege gewählt werden.
- Im Zweifel soll eine Rücksprache mit dem Transplantationszentrum erfolgen.
